# The association of disease type, pre-transplant hemoglobin level and platelet count with transfusion requirement after autologous hematopoietic stem cell transplantation

**DOI:** 10.22088/cjim.12.4.544

**Published:** 2021

**Authors:** Shabnam Tabasi, Sayeh Parkhideh, Elham Roshandel, Samira Karami, Anahita Saeedi, Ali Jabbari, Abbas Hajifathali

**Affiliations:** 1 Hematopoietic Stem Cell Research Center, Shahid Beheshti University of Medical Sciences, Tehran, Iran; 2 Department of Anesthesiology and Critical Care Medicine, Golestan University of Medical Sciences (GoUMS), Head of the Clinical Research Development Center, Gorgan, Iran

**Keywords:** Autologous, Hematopoietic stem cell transplantation, Hemoglobin, Platelet, Transfusion

## Abstract

**Background:**

Autologous hematopoietic stem cell transplantation (auto-HSCT) has become an effective treatment for a wide range of hematologic and non-hematologic diseases. Patients undergoing HSCT might require multiple platelets and red blood cell (RBC) transfusions during aplasia phase until engraftment, which could profoundly affect patients’ conditions. Identification of risk factors associated with blood product requirements could help in decreasing transfusion-related complications. We evaluated the association of disease type, pre-transplant hemoglobin level, and pre-transplant platelet count with RBC/platelet transfusion requirement after auto-HSCT.

**Methods:**

In this retrospective study, 324 patients diagnosed with multiple myeloma (MM), Hodgkin disease (HD), and non-Hodgkin lymphoma (NHL) and underwent auto-HSCT were included. The associations of disease type, pre-transplant hemoglobin level, and platelet count with post-transplant packed cell and single-/random-donor platelet transfusions were evaluated.

**Results:**

Our study results illustrated that the higher pre-transplant hemoglobin level significantly decreased the post-HSCT requirement for packed cell (IRR=0.81, [CI: 9.73-0.90], P=0.0001), while the pre-transplant platelet showed no significant relationship with platelet requirement after HSCT. HD was associated with increment in packed cell (IRR=2.04, [CI: 1.35-3.08], P=0.001) and single donor platelet (IRR=1.39, [CI: 1.09-1.78], P=0.008) requirement after transplant. The trends showed that a higher platelet level led to a lower need for platelet transfusion.

**Conclusion:**

Pre-transplant hemoglobin level could be valuable markers for predicting post-HSCT RBC requirements and might be beneficial for better management of transfusion requirements to minimize the transfusion-related complications. Patients with HD seem to be more prone to blood product requirements post-transplant.

Autologous hematopoietic stem cell transplantation (auto-HSCT) is the most common type of HSCT (57-59% of all HSCTs) for the treatment of hematologic malignancies (1-3). Platelet and packed red blood cells (RBCs) transfusion has become a standard supportive treatment in clinical practices for post-HSCT anemia, bleeding and thrombocytopenia due to bone marrow failure following conditioning regimen (2, 4, 5). There are two major applications of platelet transfusion: prophylactic platelet transfusion and therapeutic platelet transfusion. Various studies compared the benefits of these two types in treating post-HSCT complications (4, 6).

A standard threshold for the use of prophylactic platelet transfusion has been determined as platelet counts fall below 20 109/L after transplantation (7). Platelet concentration for transfusion can be provided from random donor (RD) or single donor (SD) platelet apheresis, both of which are equally safe and effective (8). Although all the transfusion units are leukodepleted, they might cause complications like allosensitization and allergic reactions. Consequently, the number of transfused units should be minimized to prevent these problems (2). 

Packed cells are transfused based on patients' hemoglobin (Hb) level as well as other clinical criteria. When a patient has a Hb level of 6 g/dL or less, there is an indication for transfusion, which continues until the patient reaches a stable hemoglobin level of 10 g/dL or more without bleeding (9-11). Packed cell transfusion has some risks for patients like alloimmune reactions, iron-overload, and infectious diseases, although the improvements in blood transfusions should never be overlooked (12). 

Hence, reducing packed cell units could decrease patients' exposure to allogeneic blood products (11). Despite a vast use of transfusion in HSCT, evidence regarding RBC and platelet transfusions and their consequences are lacking. Formerly, the transfusion was broadly prescribed (12, 13). However, given to the growing allo-immune reactions and poor prognosis of multi-transfused patients, the strategies to predict the transfusion requirements before HSCT could affect the decision making in conditioning regimen and HSCT procedure (9, 11). 

In this study, we aimed to analyze the effect of disease type, pre-transplantation hemoglobin level, and platelet count on platelet and RBC packed cell transfusion requirements. We hypothesized that low pre-transplantation hemoglobin level and platelet count as well as underlying disease could be risk factors for higher blood components transfusion requirements after autologous-HSCT.

## Methods

**Patient selection:** This retrospective study was carried out on 324 patients who underwent autologous-HSCT at Taleghani Hospital, Tehran, Iran, between March 2011 and March 2018. The required data were collected by reviewing the patients’ clinical records. Patients diagnosed with hematological disorders, including Hodgkin disease (HD), non-Hodgkin lymphoma (NHL), and multiple myeloma (MM) were included in this study. The patients with insufficient documentation, patients who were under treatment, or had been treated before (irrelevant treatments), those who had relapsed following chemotherapy, and undiagnosed patients were excluded from the study. 

Disease type (HL, NHL, MM), pre-transplant platelet count, and hemoglobin level were evaluated as risk factors. The study was approved by Shahid Beheshti University of Medical Sciences Ethics Committee with the code of IR.SBMU.REC.1398.149, and all patients provided a written informed consent.

**Stem cell Transplantation procedure:** Stem cell mobilization was induced by administering 5-10 μg/kg of G-CSF to patients for 4-5 days. Afterwards, peripheral HSCs were harvested using Spectra Optia (Terumo BCT, Lakewood, CO) and the number of CD34+ and CD3+ cells were counted by flow cytometry (Attune NxT, Invitrogen, Carlsbad, CA, USA) on day five after G-CSF treatment using PE-conjugated human anti-CD34 (EXBIO, Czech Republic) and FITC-conjugated human anti-CD3 (Beckman Coulter, Miami, FL, US).

 All multiple myeloma patients received standard myeloablative conditioning (MAC) regimen consist of Melphalan 200 mg/m2 intravenously (IV) once a day for patients younger than 65 years old and 140 mg/m2 (IV) once a day for patients older than 65 years old plus Bortezomib 1.3 mg/m2 IV in day -2 to +1 (The transplantation day was considered as day zero). Conditioning regiment for HD and NHL patients included CCNU 200 mg/m2 (IV) in day -4, Etoposide 300 mg/m2 (IV) once a day in days -3 and -2, Cytarabine 300 mg/m2 (IV) twice a day in days -3 and -2, and Melphalan 140 mg/m2 one dose in day -1. All patients received ciprofloxacin for bacterial prophylaxis, fluconazole, or itraconazole for fungal prophylaxis and acyclovir for viral prophylaxis. 

**Transfusion procedure:** All patients received random and single donor platelet concentrations and packed red cells that were ABO compatible, according to IBTO (Iranian Blood Transfusion Organization) guidelines. All transfusion units were leukocyte-reduced (<106 leukocytes). Hemoglobin level and platelet counts were measured every morning from the admission to discharge day using Sysmex XE-2100 automated blood cell counter (Sysmex, Kobe, Japan). Single and random donor platelet transfusions were administered one week after stem cell transfusion until the platelet count became higher than 20×109/L. Patients received RBC packed cells to maintain hemoglobin levels above 9 gm/dL. 

**Statistical analysis:** The variables in this study, including packed red cells and single and random donor platelet transfusions were discrete variables. A regression model was employed to evaluate the effect of disease type, pre-transplant hemoglobin level, and platelet count on packed cell and platelet transfusion requirements after transplantation. In these models, the overdispersion assumption was assessed for the selection of the proper model. Due to the existence of overdispersion for all variables, the Negative Binomial regression model was chosen. The analysis was performed with SPSS (Version 22.0). P-values less than 0.05 were assumed statistically significant.

## Results

In the present study, 324 patients with MM, HD, and NHL received autologous-HSCT in the Bone Marrow Transplantation ward at Taleghani Hospital, Tehran, Iran. The effects of disease type, pre-transplant hemoglobin level, and platelet count on transfusion requirements were assessed in these patients. The descriptive statistics of the variables for each disease as well as the clinical data of the patients are illustrated in table 1.

 The mean platelet transfusion for HD was 1.95, and 124 patients had red packed cell transfusion. The mean pre-transplant platelet count was 112865 in HD patients. In these patients, the minimum and maximum pre-transplant hemoglobin levels were 2 and 11.5, in the order given. In this study, we applied the Negative Binomial regression model to investigate the effects of outcome variables on transfusion requirements after transplantation. The results of this model for the effects of pre-transplant hemoglobin level and disease type on packed red cell transfusion requirements are shown in table 2.

The results in table 2 demonstrate that hemoglobin level and disease type significantly affected packed cell transfusion requirements. For each unit (g/dL) increase in hemoglobin, holding the disease type constant, the mean estimated transfusion units were decreased by 19%. The mean estimated transfusion units in HD patients were two times greater than MM patients, which was statistically significant.

**Figure 1:**
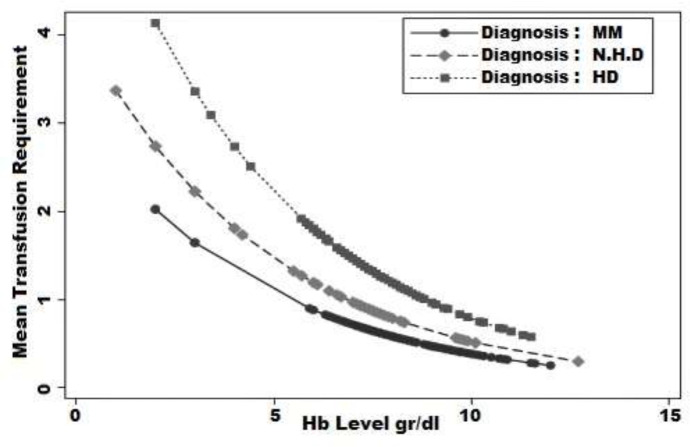
The effect of hemoglobin level on packed cell transfusion requirement for each disease. MM. Multiple Myeloma; HD. Hodgkin Disease; NHL. Non-Hodgkin Lymphoma

At the same time, the results pointed out that the mean estimated transfusion units in NHL patients, holding the hemoglobin level constant, were 1.3 times higher than MM patients, and it was not statistically significant. Figure 1 presents the predicted transfusion units based on disease type and hemoglobin level. As illustrated in figure 1, MM patients had the lowest, and HD patients had the highest transfusion requirement. Moreover, the mean transfusion units decreased by the increase in hemoglobin levels for all three groups patients.

**Table 1:** Clinical information of patients

**Variables**	**Mean SD / Median (Range) / Frequency (%)** **N=324**
**Age**	42.5313.38
**Gender**	
Male	170(52.4%)
Female	154(47.6%)
**Disease**	
Multiple myeloma	160 (49.4%)
Non-Hodgkin lymphoma	66 (20.4%)
Hodgkin disease	98 (30.2%)
**Diagnosis to transplant (day)**	419 (35-4127)
**Blood Group**	
A	116 (35.8%)
B	69 (21.3%)
AB	44 (13.6%)
O	95 (29.3)
**Disease duration (month)**	18.19.17
**Pre-transplant PLT count**	
MM	111052.90±627001.31
NHL	112865.38±68004.62
HD	106147.83±73113.26
**Pre-transplant Hb level**	
MM	7.68±2.20
NHL	6.62±2.56
HD	7.26±2.06
**PLT transfusion requirement**	
MM	1.41±1.69 / (0-11)1
NHL	1.82±1.76 / (0-6)1
HD	1.95±2.08 / (0-14)1
**Red packed cell transfusion requirement**	
MM	1.08±2.19 / (0-13)1
NHL	0.98±2.60 / (0-11)1
HD	1.56± 4.01 / (0-25)1

The platelet/ RBC transfusions’ range among patients; PLT. Platelet; Hb. Hemoglobin; MM. Multiple Myeloma; HD. Hodgkin Disease; NHL. Non-Hodgkin Lymphoma

**Table 2:** The effect of pre-transplant hemoglobin level and disease type on packed cell transfusion requirement

Pvalue	(95% CI)	SE2	IRR1	Packed cells transfusion
**0.000**	(0.73-0.90)	0.04	0.81	Hemoglobin level
				Diagnosis
**0.270**	(0.79-2.31)	0.37	1.35	NHL
**0.001**	(1.35-3.08)	0.43	2.04	HD
-	-	-	-	MM(RL3)

IRR: Incidence Rate Ratio; SE: Standard Error; RL: Reference Level; MM: Multiple Myeloma; HD: Hodgkin Disease; NHL: Non-Hodgkin Lymphoma

Table 3 indicates the pre-transplant platelet count and disease type effect on single donor platelet (SDP) and random donor platelet (RDP) transfusion requirement. There was no significant relationship between pre-transplant platelet count and single/random donor platelet transfusion requirement. The only disease type which had a significant effect on the SDP transfusion requirement was HD. Other disease types did not have any significant effect. The mean estimated SDP transfusion units in HD patients, while the platelet count remains constant, was about 1.39 times higher than MM patients. As described in table 3, the mean estimated RDP transfusion units in HD patients, while the platelet count remained constant, was 1.55 times higher compared to MM patients; however, it was not significant. For NHL patients, the mean estimated RDP transfusion was not different compared to MM patients, which was also not significant. 

**Figure 2:**
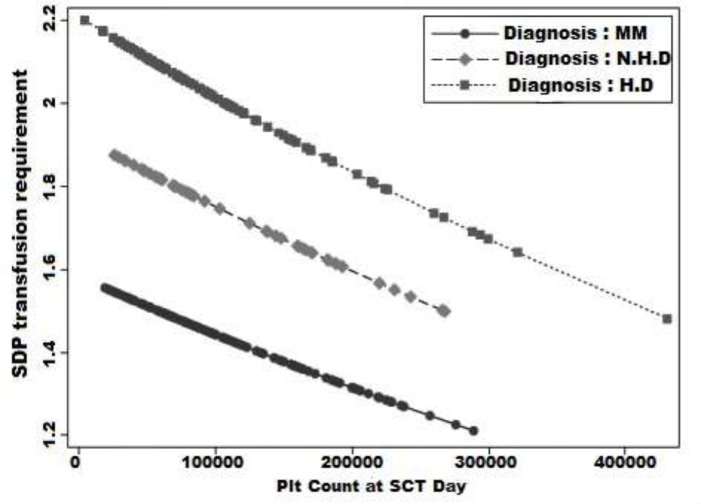
The effect of pre-transplant platelet counts on SDP transfusion requirement for each disease MM. Multiple Myeloma; HD. Hodgkin Disease; NHL. Non-Hodgkin Lymphoma

Figure 2 demonstrates the predicted requirements for single donor platelet transfusion units in each disease type based on pre-transplant platelet count. Patients with MM had the least requirement for SD PLT transfusion. Also, based on the trends that seem approximately parallel, by increasing the pre-transplant platelet, the SD platelet requirement decreased with the same rate in all three diseases. 

**Table 3:** The effect of pre-transplant platelet counts and disease type on single and random donor platelet transfusion requirement

P-value	(95%CI)	SE	IRR	
**0.282**	(0.99-1.00)	0.00	0.99	PLT count at SCT(SD)
**0.433**	(0.99-1.00)	0.00	1.00	PLT count at SCT(RD)
**0.236** **0.008** **-**	(0.88-1.67) (1.09-1.78) -	0.20 0.17 -	1.21 1.39 -	Diagnosis(SD) NHL HD MM(RL3)
**0.893** **0.187** **-**	(0.40-2.20) (0.81-2.99) -	0.41 0.52 -	0.94 1.55 -	Diagnosis(RD) NHL HD MM(RL3)

IRR. Incidence Rate Ratio; SE. Standard Error; RL. Reference Level; MM. Multiple Myeloma; HD. Hodgkin Disease; NHL. Non-Hodgkin Lymphoma SD. Single Donor RD. Random Donor

## Discussion

In the current study, we assessed the effects of disease type, hemoglobin concentration, and platelet count before autologous HSCT on the blood product transfusions requirement (Packed red cells and platelet) post-HSCT. We found that the low pre-transplant hemoglobin level is a risk factor for the RBC transfusion requirement after HSCT. Besides, the underlying disease is another risk factor for both RBC and platelet requirements. The patients with multiple myeloma need the lowest and HD patients requiring the most RBC and platelet transfusion among the three studied diseases. 

Patients undergoing HSCT need the support of blood transfusions and blood products until the engraftment time of red blood cells and platelets. Understanding the effective factors in determining the time and need for transfusion could reduce the incidence of life-threatening reactions due to over-transfusion. Since no prospective study has been conducted on the transfusion of red blood cells in patients undergoing HSCT, the American Association of Blood Banks (AABB) guidelines are mostly employed for the transfusion of red blood cells in patients undergoing HSCT. According to AABB, the blood transfusion is applied for hemoglobin level 7-8 g/dl in stable patients unless they have symptomatic anemia (14).

Comprehending the effects of prognostic factors in determining the time and need for blood product transfusion will be helpful in the establishment of supportive treatment for patients along with the prevention of life-threatening side effects of alloimmunization. In the current study, we found that the pre-transplant hemoglobin level and the underlying disease have significant effects on the RBC transfusion requirement. Our results indicated that the mean transfusions of concentrated RBC in MM patients were lower than both HD and NHL patients. Hence, MM patients need the lowest RBC transfusion, while HD and NHL might be risk factors for high RBC transfusion requirement. It was also determined that by increasing the concentration of hemoglobin in 3 groups of patients, the mean transfusions would decrease. 

The drugs for malignancies often reduce production and increase the destruction of many cells, which their functionality is essential for bleeding prevention. Besides, a low platelet count often leads to hemorrhagic disorders. Hence, platelet transfusion is a significant approach for cancer management and balancing the clotting system in patients with blood disorders and cancers. However, platelet transfusion is associated with unfavorable side effects such as alloimmunization, platelet resistance, septicemia, shock, and even death, recognizing the indication of SD/RD platelet transfusions in patients after transplant who need intensive care is necessary (15, 16).

In a Trial of Poor Performance Status Patients (TOPPS trial), those patients undergoing chemotherapy or hematopoietic stem cell transplantation were divided into two therapeutic and prophylactic groups regarding the platelet transfusion requirements. Platelet transfusion in the prophylactic group was conducted on the first day that platelet count dropped below 10 × 109/L, whereas in the therapeutic group, platelet concentrations started to be transfused upon observing the first clinical symptoms. The results revealed that the lower platelet was employed in a therapeutic group compared to the prophylactic group, although bleeding in these patients started early and lasted longer (17).

It should be noted that in TOPPS trial, 70% of the patients who underwent the autologous transplant had a lower risk of bleeding compared to allogeneic transplants. Moreover, they showed that therapeutic platelet transfusion could increase the risk of bleeding compared to prophylactic platelet transfusion in thrombocytopenic patients because of chemotherapy or HSCT. Simultaneously, therapeutic platelet transfusion could lead to a reduction in the number of platelets administered, which decreased the risk of alloimmunization (17). 

In another similar trial conducted by Wandt et al. on patients who had autologous HSCT and the ones with acute myeloid leukemia (AML), it was established that bleeding in the therapeutic group was greater than the prophylactic group. Furthermore, in the therapeutic group, six patients had cranial bleeding, while bleeding was not observed in the prophylactic group. In this study, like TOPPS trial, a significant decrease in platelet transfusion of the therapeutic group was observed (18). According to TOPPS trial findings, which illustrated that prophylactic transfusion could be utilized as the supportive treatment, by reducing the bleeding. Stanworth SJ et al. showed that the effectiveness of prophylactic platelet transfusions might differ between diseases depending on patient diagnosis and treatment (19).

Another aim of this research was to determine the influential factors in predicting the time and PLT/RD and PLT/SD transfusion requirement after autologous HSCT. Our results demonstrated that patients with HD needed more SD and RD platelet transfusions that might be due to a more intensive chemotherapy regimen before transplantation. Therefore, in this group of patients, prophylactic platelet transfusion could be more beneficial. The results showed that the estimated mean of SD platelet count in HD patients is greater than MM patients. It seems that HD patients who received more intensive chemotherapy regimens or radiation therapy prior to transplantation might have more damaged bone marrow microenvironment. Damage in the bone marrow microenvironment could probably lead to thrombocytopenia, potentially reducing the speed of platelet engraftment (13, 20).

The results of the trend showed that by increasing the pre-transplant platelet, SDP transfusion requirements decreased with the same rate for all three disease types. Comparing the SD and RD platelets identified that none of the variables under study had any significant effect on the RD platelet transfusion requirement, and all three types had the same level of requirement for RD platelet transfusions. Therefore, the underlying disease seems to be more important as a risk factor for post-HSCT platelet transfusion requirements compared to the pre-transplant platelet count. 

It is noteworthy that the requirement to blood products in HSCT patients depends on numerous factors such as age, sex, comorbidities, and previous treatments. Therefore, the patients who were under treatment, or had been treated before with irrelevant drugs, those who had relapsed following chemotherapy, and undiagnosed patients were excluded from the study. However, all the influencing factors were not excluded in the analysis and is suggested to be considered in the future investigations.

Conclusively, we found that patients with Hodgkin disease are more susceptible to RBC and platelet transfusions. Disregarding of the disease, pre-transplant low hemoglobin level is a risk factor for post-HSCT RBC transfusion requirement. The role of underlying disease on post-HSCT platelet requirement seems to be more important than the pre-transplant platelet count. When all is said and done, we assert that factors such as pre-transplant hemoglobin concentration, disease type and probably platelet count should be considered as influential factors involved in predicting the post-transplant blood transfusion and blood product requirement, so that the incidence of undesirable and life-threatening side effects could be prevented. 
